# In vitro antihelmintic effect of fifteen tropical plant extracts on excysted flukes of Fasciola hepatica

**DOI:** 10.1186/s12917-015-0362-4

**Published:** 2015-02-27

**Authors:** José Manuel Alvarez-Mercado, Froylán Ibarra-Velarde, Miguel Ángel Alonso-Díaz, Yolanda Vera-Montenegro, José Guillermo Avila-Acevedo, Ana María García-Bores

**Affiliations:** Departamento de Parasitología, Facultad de Medicina Veterinaria y Zootecnia, Universidad Nacional Autónoma de México. Cd. Universitaria, C.P. 04510 México, DF Mexico; Centro de Enseñanza Investigación y Extensión en Ganadería Tropical, Facultad de Medicina Veterinaria y Zootecnia, Universidad Nacional Autónoma de México, Km. 5.5, Carretera Federal Tlapacoyan-Martínez de la Torre, C.P. 93600 Veracruz, Mexico; Lab. de Fitoquímica, UBIPRO, Facultad de Estudios Superiores Iztacala, UNAM, Avenida de los Barrios 1, C.P. 54090 Edo. de México, Mexico

**Keywords:** Plant extracts, *Fasciola hepatica*, Anthelmintic activity, *In vitro*

## Abstract

**Background:**

Fasciolosis due to *Fasciola hepatica* is the most important hepatic disease in veterinary medicine. Its relevance is important because of the major economical losses to the cattle industry such as: reduction in milk, meat and wool production; miscarriages, anemia, liver condemnation and occasionally deaths, are estimated in billons of dollars.

The emergence of fluke resistance due to over or under dosing of fasciolides as well as environmental damage produced by the chemicals eliminated in field have stimulated the need for alternative methods to control *Fasciola hepatica*. The aim of this study was to evaluate the *in vitro* anthelmintic effect of fifteen tropical plant extracts used in tradicional Mexican medicine, on newly excysted flukes of *Fasciola hepatica*.

**Results:**

The flukes were exposed in triplicate at 500, 250 and 125 mg/L to each extract. The efficacy was assessed as the mortality rate based on the number of live and dead flukes after 24, 48 and 72 h post-exposure. The plants with anthelmintic effect were evaluated once again with a concentration of 375 mg/L in order to confirm the results and to calculate lethal concentrations at 50%, 90% and 99% (LC_50_, LC_90_, and LC_99_). Plant extracts of *Lantana camara*, *Bocconia frutescens*, *Piper auritum, Artemisia mexicana* and *Cajanus cajan* had an *in vitro* anthelmintic effect (P <0.05). The LC_50_, LC_90_ and LC_99_ to *A. mexicana*, *C. cajan* and *B. frutescens* were 92.85, 210.44 and 410.04 mg/L, 382.73, 570.09 and 788.9 mg/L and 369.96, 529.94 and 710.34 mg/L, respectively.

**Conclusion:**

It is concluded that five tropical plant extracts had promising anthelmintic effects against *F. hepatica*. Further studies on toxicity and *in vivo* biological evaluation in ruminant models might help to determine the anthelmintic potential of these plant extracts.

## Background

Fasciolosis caused by *Fasciola hepatica* has a worldwide distribution affecting cattle, sheep, goats, pigs, horses, rabbits and humans as well. It causes major economical losses to the cattle industry (estimated in billons of dollars) by decreasing milk and/or meat production, low reproductive efficiency, liver seizures in slaughterhouses, high costs to control parasitism and deaths [1,2].

The control of this disease has been based on the application of anthelmintics, but due to the development of resistance it seems that the efficacy of some chemical drugs has decreased [3,4]. The use of plants with anthelmintic activity may be an alternative to fluke control, given the great diversity of ecosystems. The opportunity of finding bioactive compounds with anti-fluke properties significantly increases because, secondary metabolites (SM) are the most important compounds as new alternatives for parasite control. Some SM such us alkaloids, saponins, skimmiarins A and C, tannins, flavonoids, terpenes (mono, di and sesquiterpenes) have been shown to be active against a wide range of parasites [5]. Recent studies have reported the anthelmintic effect of plants such as *Artemisia mexicana, Mentha piperita, Achillea millefolium, Allium sativum, Piper nigrum, and Carica papaya* with parasiticidal effects against *F. hepatica* [6-8].

Veracruz is the Mexican state with the highest livestock production in the country [9] and parasitic illnesses are the main threat to grazing bovines in this region. Because of the great diversity of ecosystems, the native vegetation of Veracruz has a wide variety of plant species (containing variable levels of SM) which potentially could be used as a fascioliscide. However, studies to evaluate the effect of plants with possible anthelmintic properties against *F. hepatica* in the area have been not carried out. The aim of the present study was to evaluate the anthelmintic effect of fifteen plants extracts from Veracruz, Mexico.

## Methods

### Plant material

Fresh leaves (700 g) of *Acacia cornigera* (2147 IZTA), *Acacia farnesiana* (2164 IZTA), *Artemisia absinthium* (2155 IZTA), *Artemisia mexicana* (2156 IZTA), *Bocconia frutescens* (2153 IZTA), *Cajanus cajan* (2164 IZTA), *Cordia spp*, *Hibiscus rosa – sinensis* (2149 IZTA), *Lantana camara* (2160 IZTA), *Leucaena diversifolia* (2169 IZTA), *Melia azedarach* (2161 IZTA), *Mentha sp* (2163 IZTA), *Ocimum basilicum* (2154 IZTA), *Piper auritum* (2165 IZTA) and *Teloxys ambrosioides* (2157 IZTA) were collected from villages in Veracruz, Mexico.

Prior to the beginning of this trial, samples of different plants were collected and identified by Dr. Edith López Villafranco of the IZTA Herbarium at the Facultad de Estudios Superiores Iztacala for the purpose of authenticating them. A voucher specimen was deposited in the IZTA herbarium for future reference (a reference number was assigned). The plants were chosen based on the traditional practices [10-12]; moreover reports of other authors [7,13-15] and interviews with local people have shown to be effective in finding remedies against other parasites.

### Extraction procedure

Extraction procedures were undertaken in the phytochemistry laboratory of FES Iztacala and the evaluation of in vitro anthelmintic efficacy was carried out in the laboratory of experimental chemotherapy of the parasitology department, (FMVZ-UNAM).

The leaves of each plant (100 g) were dried in an oven for three days at 60°C, ground into powder and sequentially extracted with hexane, ethyl acetate and methanol. The extracts were filtered and successively concentrated. Each extract was concentrated under low pressure at low temperature and revolutions per minute (RPM) as follows: 1) hexane, at 60°C, 50 RPM, 2) ethyl acetate, at 78°C,60 RPM and 3) methanol, at 65°C, 90 RPM using a rotaevaporator [16,17]. The plant extracts were kept in the dark at 4°C until tested.

### Bioassays

To determine the antihelmintic effect of the 15 plant extracts on the mortality of excysted flukes a series of in vitro experiments were undertaken. Newly excysted flukes were obtained by the artificial excysment of *F. hepatica* metacercariae following the methodology described by Ibarra and Jenkins [18].

### Formulation of plant extracts for screening

All compounds were formulated as follows: 500 mg of the compound were placed in a screw-capped 15 ml Eppendorf® tube to which 0.1 ml of methanol were added to dissolve the extract. Then two fold dilutions using distilled water were made to prepare concentrations of 500, 250 and 125 mg/L.

Plant extracts were placed in NUNC® culture dishes. Each well contained 1.6 mL of RPMI-1640® of the culture medium, 0.2 mL of solubilized extract and 0.2 ml containing 10 liver flukes. Four wells were used as untreated controls, three containing only a complete medium (RPMI-1640®), the last one containing a culture medium and 0.2 ml of methanol. In addition there were four more wells containing triclabendazole (SOFOREN®, Novartis) at a 10 and 50 mg/L, respectively. Each test remained incubated at 37°C for four days under a 5% CO_2_ atmosphere; each experiment was replicated three times.

The plant extracts with in vitro anthelmintic efficacy higher than 80% were re-evaluated twice in order to confirm the results, and a concentration of 375 mg/L was added to calculate the lethal concentration to kill 50%, 90% and 99% of the flukes (LC_50_, LC_90_ and LC_99_). All procedures were performed under aseptic conditions using a laminar flow hood.

### Test interpretation

The flukes under study were examined at 24, 48 and 72 hours post-exposure. Activity was measured by comparing the survival of the treated flukes relative to those of the control group. At each evaluation time, these flukes without motility were considered as dead.

### Efficacy measurement

The effectiveness of the plant extracts was assessed with the following formula [19]:

$$ \mathrm{Efficacy}\left(\%\right) = \frac{\mathrm{No}.\ \mathrm{of}\ \mathrm{flukes}\ \mathrm{alive}\ \mathrm{in}\ \mathrm{control}\ \mathrm{group}-\mathrm{No}.\ \mathrm{of}\ \mathrm{flukes}\ \mathrm{alive}\ \mathrm{in}\ \mathrm{treated}\ \mathrm{group}}{\mathrm{No}.\ \mathrm{of}\ \mathrm{flukes}\ \mathrm{alive}\ \mathrm{in}\ \mathrm{control}\ \mathrm{group}} \times 100 $$

When an extract showed an in vitro efficacy greater than 80%, it was considered to possess fascioliscide activity.

### Phytochemical screening

The active extracts were subjected to phytochemical analysis to determine the presence of SM groups following standard published protocols [20,21].

### Statistical analyses

A Kruskal-Wallis test, P <0.05 was used to determine significant differences [22] and a PROBIT test was performed with POLO PLUS [23] to determine the LC_50_, LC_90_ and LC_99_ of the extracts that showed in vitro fascioliscide efficacy.

## Results

### Efficacy of the extracts

The flukes placed in the control wells remained alive and healthy throughout all the tests. From 15 plants evaluated (Table 1), five plant extracts at different dose levels effectively killed *Fasciola hepatica* (P <0.05). At a dose of 500 mg/L, *C. cajan, L. camara* and *P. auritum* had an efficacy of 100%, while *B. frutescens* and *A. mexicana* had a 100% efficacy at a dose of 125 mg/L.

**Table 1:** **In vitro anti-fluke effectiveness of fifteen plant extracts**

**Plant extract**		**Reference control (%)** ^d^		**Untreated control (%)** ^e^	**Efficacy (%)** ^c^		
		**10 mg/L**	**50 mg/L**	**0 mg/L**	**125 mg/L**	**250 mg/L**	**500 mg/L**
*A. cornígera*	n = 10	100^a^	100^b^	0^a^	0^a^	0^a^	0^a^
*C. cajan*		100^a^	100^b^	0^a^	0^a^	0^a^	100^b^
*A. farnesiana*		100^a^	100^b^	0^a^	0^a^	0^a^	0^a^
*L. camara*		100^a^	100^b^	0^a^	0^a^	0^a^	100^b^
*H. rosa - sinensis*		100^a^	100^b^	0^a^	0^a^	0^a^	0^a^
*B. frutescens*		100^a^	100^b^	0^a^	10 ± 0.1^a^	100^b^	100^b^
*M. azedarach*		100^a^	100^b^	0^a^	7 ± 0.11^a^	7 ± 0.11^a^	13 ± 0.11^a^
*L. diversifolia*		100^a^	100^b^	0^a^	0^a^	0^a^	0^a^
*C. spp*		100^a^	100^b^	0^a^	0^a^	0^a^	0^a^
*C. ambrosioides*		100^a^	100^b^	0^a^	0^a^	0^a^	0^a^
*P. auritum*		100^a^	100^b^	0^a^	0^a^	0^a^	100^b^
*M. sativa*		100^a^	100^b^	0^a^	0^a^	0^a^	0^a^
*A. absinthium L.*		100^a^	100^b^	0^a^	0^a^	0^a^	0^a^
*O. basiliam*		100^a^	100^b^	0^a^	0^a^	0^a^	0^a^
*A. mexicana*		100^a^	100^b^	0^a^	100^b^	100^b^	100^b^

^a,b^A different letter between columns indicates statistically significant differences. Significant at p < 0.05 level. Control—nil mortality.

^c^Average of three replicates ± standard deviation.

^d^Triclabendazole, average of three replicates ± standard deviation.

^e^Destilled water, average of three replicates ± standard deviation.

The five extracts showing *in vitro* anthelmintic activity greater than 80% are indicated in Table 2. These were evaluated for a second time including a concentration of 375 mg/L to determine LC_50_, LC_90_ and LC_99_. The results were consistent with the previous one described above.

**Table 2:** **Second assessment of anti-fluke effectiveness of five plant extracts**

**Plant extract**		**Reference control (%)** ^d^		**Untreated control (%)** ^e^	**Efficacy (%)** ^c^			
		**10 mg/L**	**50 mg/L**	**0 mg/L**	**125 mg/l**	**250 mg/l**	**500 mg/l**	**500 mg/l**
*A. mexicana*	n = 10	100^a^	100^b^	0^a^	93 ± 0.06^b^	100^a^	100^a^	100^a^
*B. frutescens*		100^a^	100^b^	0^a^	0^a^	100^b^	100^b^	100^b^
*L. camara*		100^a^	100^b^	0^a^	0^a^	0^a^	93 ± 0.06^b^	100^b^
*P. auritum*		100^a^	100^b^	0^a^	0^a^	0^a^	83 ± 0.06^b^	100^b^
*C. cajan*		100^a^	100^b^	0^a^	0^a^	0^a^	93 ± 0.06^b^	93 ± 0.06^b^

^a,b^A different letter between columns indicates statistically significant differences. Significant at p < 0.05 level. Control—nil mortality.

^c^Average of three replicates ± standard deviation.

^d^Triclabendazole, average of three replicates ± standard deviation.

^e^Destilled water, average of three replicates ± standard deviation.

**Figure 1:**
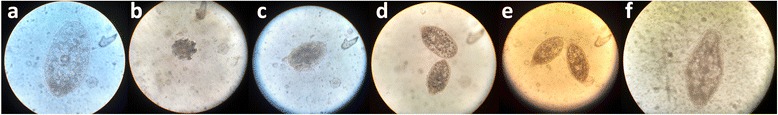
**Flukicide activity of plant extracts. a**. Untreated control flukes. **b**. Flukes treated with *L. camara* extract 72 hrs post exposition. Dead flukes being severely affected in the tegument and internal organs. **c**. Flukes treated with *A. mexicana* extract 72 hrs post exposition. Flukes showed no motility and internal changes. **d**. Flukes treated with *P. auritum* extract 72 hrs post exposition. Flukes showed no motility and no internal changes. **e**. Flukes treated with *C. cajan* extract 72 hrs post exposition. Flukes showed no motility and no internal changes. **f**. Flukes treated with *B. frutescens* extract 72 hrs post exposition. Flukes showed no motility, but presented internal changes and litghtly affected tegument.

Figure 1 shows the flukicide activity before and after exposition with some plant extracts at 40*×*.

### Lethal concentration estimates at 50%, 90% and 99% for exposed flukes to plant extracts

The slopes LC 50, LC 90 and LC 99% in *A. mexicana*, *C. cajan* and *B. frutescens* tested plant extracts are shown in Table 3. *A. mexicana* showed significantly lower LC_50_, LC_90_ and LC_99_ than *C. cajan* and *B. frutescens,* but it was not possible to calculate LC for *P. auritum* and *L. camara* due to their high efficacy (100%), but it was possible to be done in the two higher doses.

**Table 3:** **Lethal concentration estimates from plant extracts with anthelmintic efficacy in vitro**

**Plant extract**	**LC**_50_ **(mg/L)**	**LCL-UCL**	**LC**_90_ **(mg/L)**	**LCL-UCL**	**LC**_99_ **(mg/L)**	**LCL-UCL**	**SD**	***×*** ^2^ **(df = 10)**
*A. mexicana*	92.85	42.16-124.50	210.44	166.78-306.78	410.04	288.46-1135.26	±2.197	5.893
*C. cajan*	382.73	327.13-444.12	570.09	479.89-908.48	788.9	603.92-1768.3	±3.653	15.258
*B. frutescens*	369.96	318.77-419.83	529.94	457.78-748.36	710.34	567.74-1298.47	±3.813	14.702

LC50 — lethal concentration that kills 50% of the exposed flukes, LC90 — lethal concentration that kills 90% of the exposed flukes, LC99 — lethal concentration that kills 99% of the exposed flukes, UCL: upper confidence limit; LCL: lower confidence limit, SD: standard deviation. *×* ^2^ — Chi-square; df: degree of freedom. Significant at p < 0.05 level.

### Phytochemical screening

Table 4 shows that most crude extracts contain MS such as alkaloids, phenolic compounds as well as coumarins, flavanones and flavonoids. Furthermore, sesquiterpen lactones, steroids, triterpenes and glycosides were also detected.

**Table 4:** **Results of phytochemical screening**

**Colorimetric reaction**	**Plant extract**				
	***L. camara***	***B. frutescens***	***P. auritum***	***C. cajan***	***A. mexicana***
Phenolic compounds (FeCl3)	++	+	++	++	+
Coumarins (UV)	--	++	--	--	+
Flavanones (NH3)	+ Yellow	--	+ Yellow	+ Yellow	+ Yellow
Flavonoids (Shinoda)	--	--	+ Red	+ Orange	+ Red
Sesquiterpene lactones (Baljet)	+	--	+	+	+
Alkaloids (Meyer)	+++	+++	+++	+++	+++
Alkaloids (Dragendorff)	+++	+++	+++	+++	+++
Steroids and triterpenoids (Liberman, Burchard)	++	++	+	+	+
Glycosides (α-naphtol)	--	--	--	--	+

Symbology: −− negative; + weak positive; ++ positive; +++ strong positive.

## Discussion

Plant extracts currently represent a potencial alternative for the effective control of fasciolosis in domestic ruminants. However, since this area has been explored only to a limited extent, there is a manifest need to carry out new research to determine their potential against *F. hepatica*.

Jeyathilakan et al. [24] evaluated on *Fasciola gigantica* adults the efficacy of ethno-medicinal plant aqueous extracts such as *Allium sativum*, *Lawsonia inermis*, and *Opuntia ficus indica* in vitro in comparison with Oxyclozanide with efficacies from 40 – 100%. Jeyathilakan et al. [25] evaluated the essential oils of *Cymbopogan nardus* and *Azadirachta indica*. The results indicate that the essential oil of citronella showed a potential anthelmintic activity (100%) whereas neem oil did not show any significant effect. Their results indicated the potential for developing herbal-based anthelmintics to control *F.gigantica* in livestock*.*

In this study, five plant extracts showed fascioliscide activity: *A. mexicana*, *B. frutescens*, *L. camara*, *P. auritum* and *C. cajan* (P <0.05). Recent studies have reported that, at the same concentrations used in our study, *A. mexicana* extract had an anthelmintic of efficacy 100% [19,26]. The latter findings show that at the doses tested, *A. mexicana* has an intrinsic anti-fluke activity; it also indicates that this extract may be an alternative to the chemical control of *F. hepatica* only after evaluation and *in vivo* toxicity studies. In this regard, studies by Ibarra-Moreno et al. [27] in CD1 mice demonstrated that the *A. mexicana* extract had no toxicity in renal or liver tissue.

To our knowledge, this is the first report of the anthelmintic effect of *P. auritum*, *B. frutescens* and *C. cajan* against *F. hepatica*. Although these plants have not been evaluated against trematodes before, they are found to possess some interesting and additional positive characteristics which deserve to be considered for future *in vivo* studies. For example, Ghanem et al. [28] reported a protective and an antioxidant effect in the plants of the Piperaceae family as well in *P. auritum* with cultured hepatocytes of mice. In addition, Estrada et al. [29] mention that acute toxicity tests show that the intake of extracts of different polarities of *P. auritum* involves no health risks. Kundu et al. [30] have also found in the *C. cajan* extract a hepato protective effect on mice. Since there are no reported toxic effects of these plants, it is possible to obtain a similar *in vivo* effect by direct administration to ruminants.

Up to now there have been no reports of anti-fluke effectiveness for *L. camara* despite its well – known toxicity in cattle and sheep. This is the first report of *in vitro* anthelmintic activity in the *L. camara* extract. However, it is necessary to consider the undesirable effects such as photosensitivity and liver disorders that are caused in the animals that consume this plant. If this plant demonstrates great anthelmintic activity in continued studies, there will be sufficient reason for further study in order to identify the causal agents responsible for this toxicity. It is, therefore, convenient to find other species of *Lantana spp* that have no toxicity reports [31].

Secondary metabolites such as alkaloids, terpenes, tannins or flavonoids contained in crude plant extracts have been related to parasiticidal activity [32-35]. Nevertheless, since these are not the only compounds that these and other plant species possess, it would be wrong to discard the effect of other bioactive compounds. Hence, it is necessary to determine the chemical composition of the extracts that show anthelmintic efficacy. Interestingly, all extracts gave a positive reaction for alkaloids. The literature shows reports of the presence of these compounds in *L. camara* [36], *B. frutescens* [37], and *P. auritum* [38], but not in *C. cajan* and *A. mexicana*. It is likely that the positive reactions in the latter species are due to the presence of nitrogen compounds such as amino acids or other amines of a non-alkaloid origin. These alkaloids are probably responsible for the biological activity; however, there are reports of non-nitrogenous substances isolated from these plants with biocide activity as well as pentacyclic triterpenoids isolated from *L. camara* [39] and sterols and sesquiterpene lactones isolated from *C. cajanus* [40] and *A. mexicana* [41], respectively.

Consequently, the present study represents preliminary information for the continuing research to demonstrate whether the data obtained can be amplified or not in order to get their SM to determine finally whether it is one SMs or a combination of SM responsible for fascioliscide activity.

## Conclusion

Of the fifteen extracts tested, five showed promising in vitro fascioliscide efficacy, thus indicating that they could possibly be strong candidates for further biological and toxicological analyses aimed at demonstrating their real potential for liver fluke control in ruminants.
